# Metastatic Colon Cancer Presenting as Gallbladder Perforation: Highlighting the Diagnostic Challenges and Clinical Implications

**DOI:** 10.14309/crj.0000000000001167

**Published:** 2023-10-19

**Authors:** Sagar Nagpal, Taif Khattak, Sindhu C. Pokhriyal, Salma Nisar, Amro Daoud, Puneet Goenka

**Affiliations:** 1 Department of Internal Medicine, East Tennessee State University, Johnson City, TN; 2 Department of Internal Medicine, Interfaith Medical Center, Brooklyn, NY; 3 Department of Pathology, East Tennessee State University, Johnson City, TN; 4 Department of Gastroenterology, East Tennessee State University, Johnson City, TN

**Keywords:** metastatic colon cancer, gallbladder perforation, immunohistochemical staining, histopathology

## Abstract

Gallbladder perforation is an uncommon occurrence that demands prompt surgical intervention, typically observed in the context of acute cholecystitis. In this article, we present an extraordinary case of gallbladder gangrene and perforation, originating from metastasis of colon cancer. The patient's presentation included an incidental discovery of colon cancer, which was indicated by histopathology of the gall bladder. This case report aims to shed light on the intricate relationship between gallbladder pathology and metastatic colon cancer, emphasizing the need for vigilant evaluation and comprehensive management strategies.

## INTRODUCTION

Gallbladder perforation, predominantly observed in acute cholecystitis, is a grave condition with a high mortality rate. Typically, it is recognized as a complication of gallbladder inflammation. However, metastasis from colon cancer leading to gallbladder perforation represents an extremely rare occurrence.

While liver metastasis is a well-known phenomenon in colorectal cancer (CRC), metastasis to the gallbladder from the colon is a relatively uncommon but clinically significant event. The occurrence of gallbladder metastasis from colon cancer is estimated to be approximately 0.2%–2% of all gallbladder malignancies.^1^ Of note also is the fact that in approximately 25% of patients with CRC, metastatic disease is present upon presentation (stage IV), and another 18% of individuals develop metastases metachronously after the initial CRC has been treated.^2^ Metastatic CRC (mCRC) primarily arises in the liver and lungs. Rare metastatic locations include the gallbladder in 0.8% of cases.^2^ Colorectal cancer has an almost singular and unique ability to migrate along epithelial surfaces and grow intraductally, thereby mimicking primary neoplasms of the gallbladder, cholangiocarcinoma, and bladder carcinomas.^3^ Identifying metastatic disease from either an established primary or a new primary at an uncommon location is essential. This plays a critical role in shaping therapeutic approaches and accurately predicting prognosis.^4^

## CASE REPORT

We present the case of a 78-year-old man with chronic obstructive pulmonary disease, obstructive sleep apnea, atrial fibrillation, and type 2 diabetes mellitus, who initially presented with persistent diffuse abdominal pain, accompanied by nausea, vomiting, and unintentional weight loss. On admission, the patient was hemodynamically unstable and necessitated vasopressor support. Physical examination revealed noticeable scleral icterus and diffuse tenderness, as well as rigidity of the abdomen.

Laboratory investigations revealed leukocytosis with a white blood cell count of 12.4 × 10^9^/L (normal 3.5–10.5 × 10^9^/L) and elevated bilirubin level of 13.6 mg/dL (normal range 0.2–1.1 mg/dL). The lactic acid level was elevated at 2.9 mmol/L (normal 0.5–2.0 mmol/L). Broad-spectrum antibiotics were initiated to treat the suspected sepsis.

**Figure 1.**
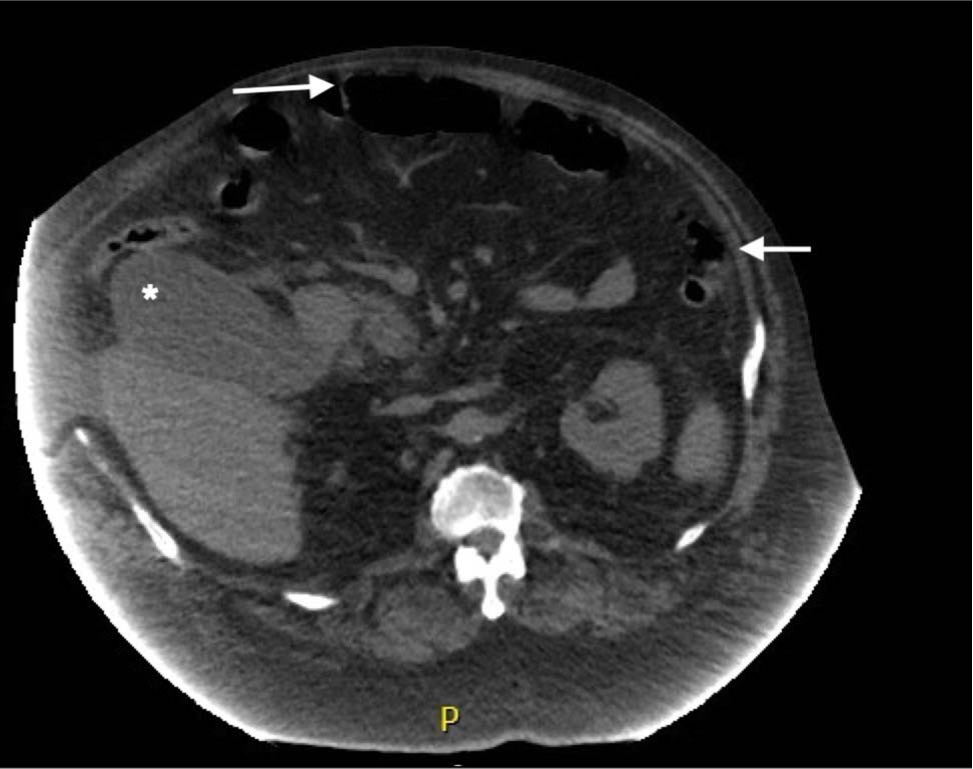
Abdominal computed tomography scan. The axial section reveals the presence of free intraperitoneal air, accompanied by the notable presentation of a distended and edematous gallbladder. Arrows pointing toward free intraperitoneal air. Asterisk showing distended and edematous gallbladder.

Imaging studies, including an abdominal-pelvic computed tomography, demonstrated the presence of small free intraperitoneal air, suggestive of a perforated bowel (Figure 1). Adenopathy was observed within the gastrohepatic ligament and periaortic area, along with bilateral iliac adenopathy. Furthermore, the patient exhibited a distended and edematous gallbladder devoid of gallstones. Additional findings from ultrasound revealed perihepatic fluid collection with septations.

The patient's condition further deteriorated because of hypotension, sepsis, and acute liver injury, with acute kidney injury resulting in elevated creatinine levels (2.9 mg/dL from a baseline of 1.4 mg/dL). Subsequent laparotomy revealed the presence of bilious ascites throughout the abdomen, with significant inflammation noted in the right paracolic gutter. The surgical intervention involved the removal of scattered fibrinous debris and adherent omentum from the lower edge of the liver. The gallbladder appeared enlarged and gangrenous with surrounding bilious drainage. Subtotal cholecystectomy and drainage were performed, and necrotic tissue was observed in the gallbladder fossa liver bed, along with inflammation in the duodenum, colon, and liver.

**Figure 2.**
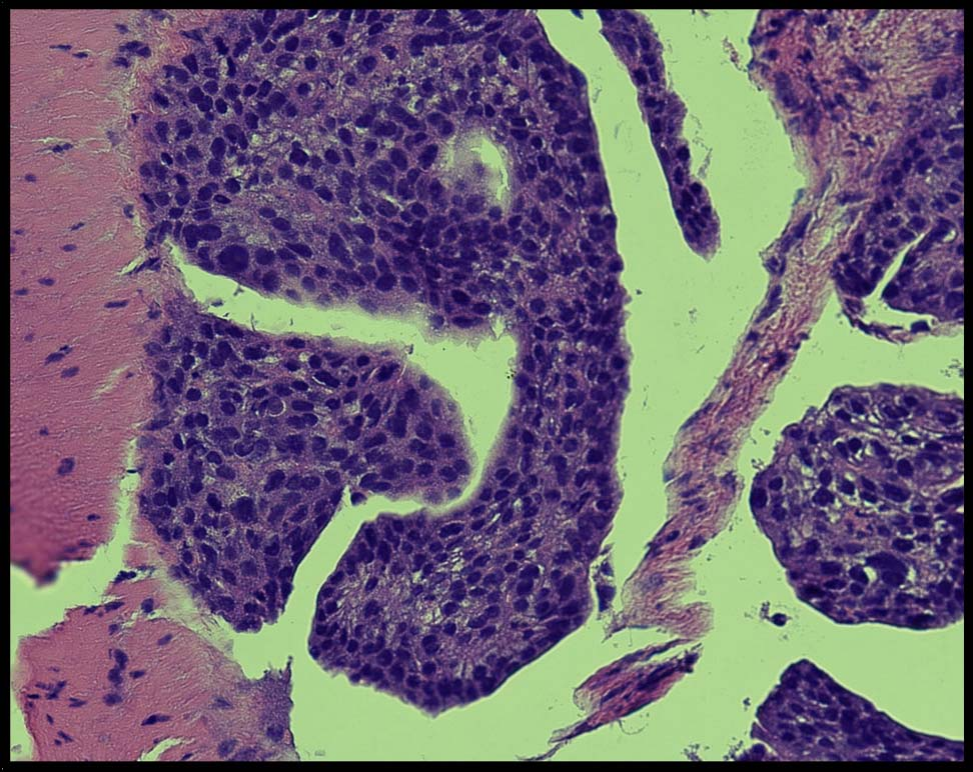
Gallbladder specimen (magnification: 400×). Hematoxylin-eosin staining of the gallbladder shows a prominent inflammatory infiltrate that occupies the entire serosa surface with abundant adenocarcinoma within lymphatic channels.

**Figure 3.**
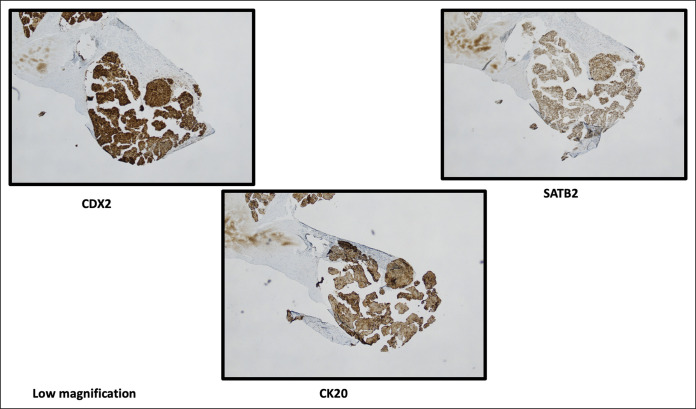
Immunoperoxidase stains show the tumor to be positive for CK20, CDX2, and SATB2, which strongly points to a gastrointestinal tract primary.

Histopathological examination of the gallbladder specimens revealed adenocarcinoma within serosal lymphatic channels, accompanied by gangrenous cholecystitis and prominent acute serositis (Figure 2). Immunoperoxidase stains turned out to be positive for cytokeratin 20 (CK20) and CDX2 while testing negative for CK7, arginase, HAS, thyroid transcription factor 1, and napsin A (Figure 3). These findings strongly suggested gastrointestinal tract primary cancer, specifically colon cancer, as evidenced by the absence of CK7, arguing against lung and hepatocellular lesions.

**Figure 4.**
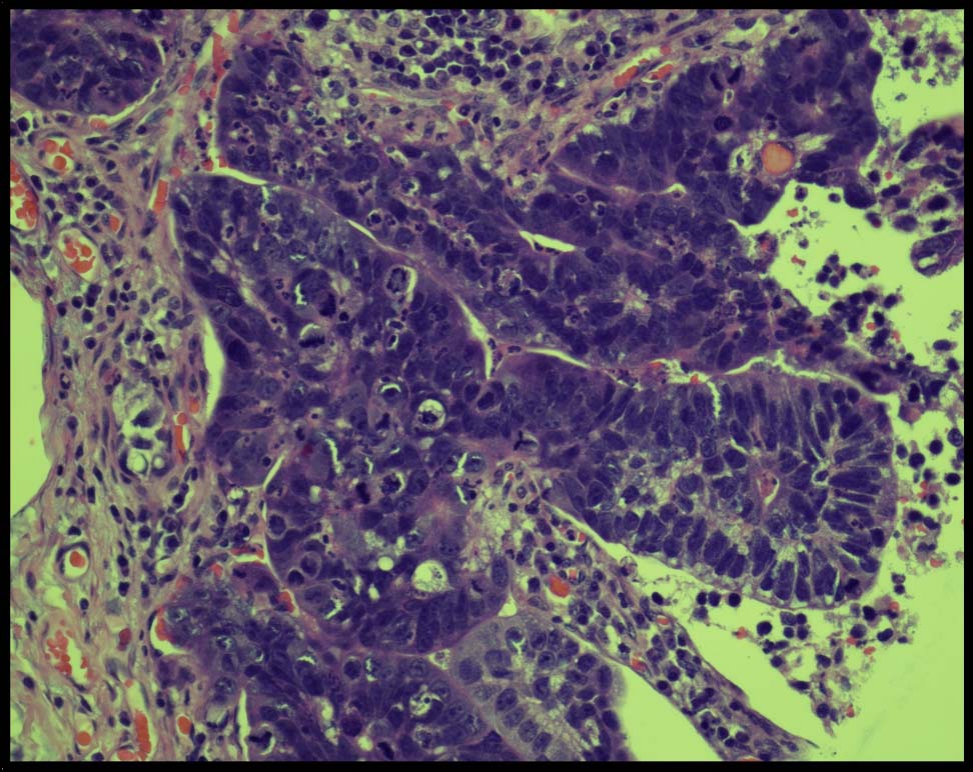
Small bowel biopsy (magnification: 400×). Hematoxylin-eosin staining of the small bowel biopsy demonstrates malignant epithelial nests and glands involving the serosal surface and bowel muscle. The neoplastic cells have enlarged hyperchromatic and pleomorphic nuclei with variable nucleoli, visible mitoses, and apoptotic debris.

Postoperatively, the patient's condition continued to worsen, necessitating vasopressor and ventilatory support for 8 days. Liver function tests revealed a significant elevation in a cholestatic pattern, with a total bilirubin of 14.9 mg/dL. Subsequently, an abdominal drain was inserted, and an ileostomy loop was created. A small bowel biopsy was obtained this time which confirmed the presence of malignant epithelial nests and glands involving the serosal surface and bowel muscle. Lymphovascular space invasion was also observed in certain areas (Figure 4). The immunophenotypic analysis demonstrated the expression of CK20, CDX2, and SATB2 while Mucicarmine staining was negative for specific cytoplasmic vacuoles. The neoplastic cells were negative for CK7, further supporting the diagnosis of adenocarcinoma and indicating a primary colon tumor.

Despite continued management, the patient's condition deteriorated further because of the advanced malignancy. After a goals-of-care discussion, the patient chose to receive hospice care.

## DISCUSSION

Metastasis to the gallbladder from the colon is a rare occurrence, and diagnosing it can be challenging because of its rarity and nonspecific symptoms. Radiological imaging plays a crucial role in identifying gallbladder lesions and their origin, although distinguishing between primary gallbladder malignancies and metastatic lesions based on imaging characteristics alone can be difficult.^5,6^ Abdominal pain from cholecystitis caused by cystic duct obstruction is the most common clinical presentation of metastatic gallbladder disease. Previous cases have reported colon cancer mimicking acute cholecystitis, suggesting that local inflammation can lead to acute acalculous cholecystitis that mimics gallbladder cancer.^6,7^

Histopathological examination remains the gold standard for confirming the presence of metastatic lesions in the gallbladder. Tissue biopsy with immunohistochemistry is often used to differentiate metastatic lesions from primary gallbladder malignancies.^8,9^ Specific markers, such as CK20 and CDX2, are commonly used to confirm colorectal origin.^2,10^ Although gallbladder tumors typically exhibit positivity for both CK7 and CK20, the presence of CK7-negative/CK20-positive cells with CDX2 positivity is indicative of a possible colorectal primary origin.^10^

Treatment decisions for metastasis to the gallbladder from the colon depend on various factors and require a multidisciplinary approach. Surgical resection, including cholecystectomy with or without additional hepatic resection, may be considered in selected cases with localized disease and favorable patient characteristics.^11,12^ Systemic chemotherapy is an option for advanced or unresectable cases. This includes combination regimens such as 5-fluorouracil, leucovorin, and oxaliplatin or 5-fluorouracil, leucovorin, and irinotecan or targeted drugs such as bevacizumab.^12,13^

Metastasis to the gallbladder from the colon generally carries a poor prognosis, indicating an advanced stage of colon cancer with limited treatment options. However, survival outcomes can vary depending on various factors, such as the extent of metastatic involvement, response to treatment, and patient-specific characteristics. Long-term survival can be achieved in select cases with early detection, aggressive surgical resection, and effective systemic therapies.^12,13^

Further research and case studies are needed to better understand optimal treatment strategies and prognostic factors associated with this rare clinical scenario.

## DISCLOSURES

Author contributions: S. Nagpal: conceptualization, data collection, and writing the original draft. T. Khattak and SC Pokhriyal: literature review and editing the manuscript. S. Nisar: pathological analysis, immunohistochemical staining, manuscript review and editing. A. Daoud: supervision, validation, manuscript review and editing. P. Goenka: final approval of the version to be published. All authors have read and approved the final manuscript. S. Nagpal is the article guarantor.

Financial disclosure: None to report.

Informed consent was obtained for this case report.
